# An experimental study on HRM combined with multiplex PCR for the identification of single and mixed diatom species

**DOI:** 10.1016/j.isci.2026.117909

**Published:** 2026-10-15

**Authors:** Yunchao Zhou, Yiwei Liu, Bin Cong, Jianhua Chen, Jianqiang Deng

**Affiliations:** 1 Key Laboratory of Tropical Translational Medicine of Ministry of Education, School of Basic Medical Sciences, Hainan Academy of Medical Sciences, Hainan Medical University, Haikou, Hainan Province 571199, China; 2 Department of Forensic Medicine / Hainan Provincial Tropical Forensic Engineering Research Center / Hainan Provincial Tropical Forensic Academician Workstation, School of Basic Medical Sciences, Hainan Medical University, Haikou 571199, P.R. China; 3 Department of Forensic Medicine, Hebei Medical University, Shijiazhuang, China

**Keywords:** high-resolution melting curve, multiplex PCR, diatom, primer, species identification

## Abstract

Diatoms are research subjects in both ecological environments and forensic science. The identification of mixed diatom samples has always been a challenging issue. Multiplex polymerase chain reaction-high-resolution melting (PCR-HRM) provides a potential solution, but the effectiveness of this method in analyzing single and mixed diatom samples remains unclear. This study focuses on eight species of marine diatoms, identifying and designing corresponding primers to preliminarily explore the HRM technique combined with multiplex PCR for species identification of marine diatoms. By using two primer combinations, we achieved rapid identification of single diatoms. The experiments revealed that the HRM technique showed reduced identification capability for mixed diatom samples, whereas the multiplex PCR-HRM technique could enhance the identification accuracy for mixed diatom samples. By using a mixture of three primers, we identified four species of diatoms in mixed samples. This method can improve the identification capability of mixed diatom samples and provides a new approach for diatom species identification.

## Highlights

• Screened and validated 15 specific primer pairs using eight marine diatom species
• HRM analysis with two primer pairs rapidly identifies single diatom species
• Conventional HRM has low resolution for mixed diatom samples, yielding fused peaks
• Multiplex PCR-HRM partially improves mixed diatom species discrimination

## Graphical abstract

**Figure:**
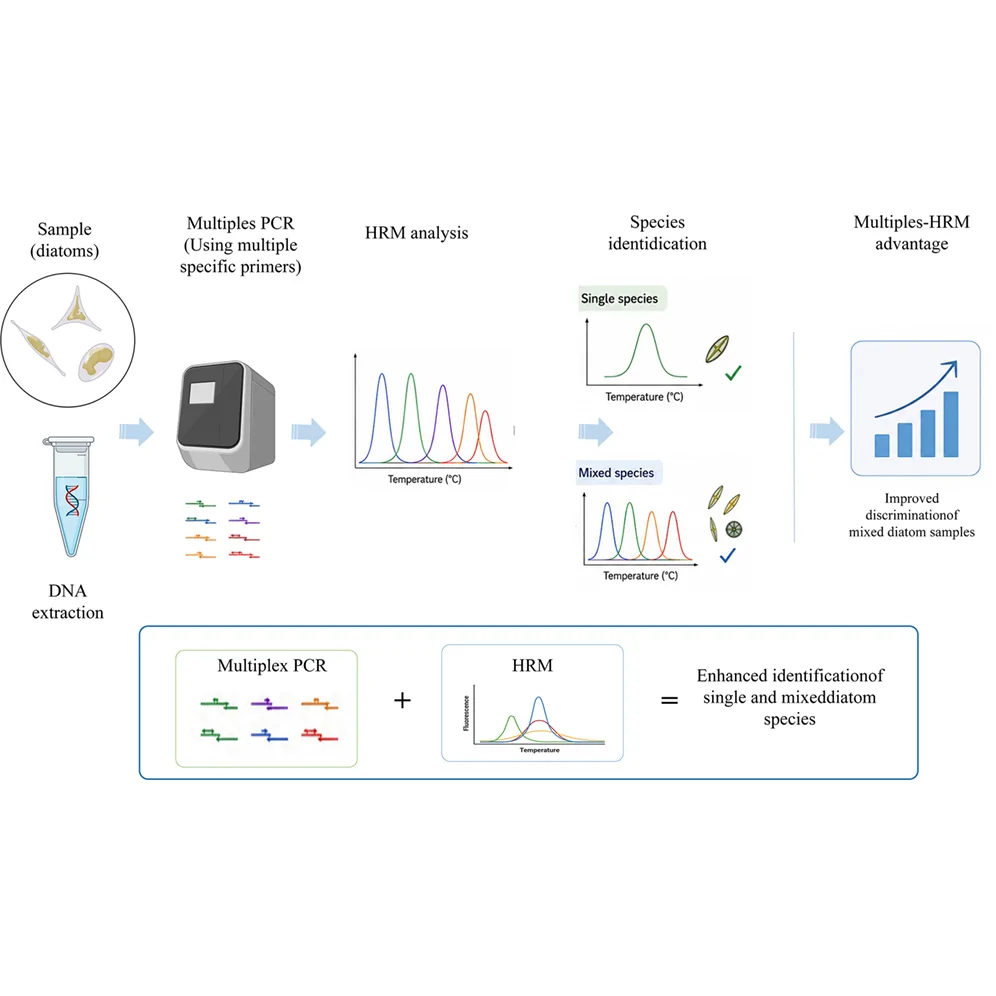

## Teaser

Biological sciences; Biotechnology

## Introduction

Diatoms are a type of aquatic unicellular plants with numerous species, widely distributed in various natural water bodies such as rivers and oceans.^1^^,^^2^ Marine diatoms play a crucial role in marine ecosystems. They are not only a food source for many marine organisms but also absorb carbon dioxide through photosynthesis and sequester a portion of carbon within their biomass. They play a key role in the global carbon cycle and biogeochemical cycles.^3^^,^^4^^,^^5^ Meanwhile, the species and quantity of diatoms can also reflect the health status of the marine ecosystem.^6^^,^^7^ Not only that but diatoms also hold significant research value in forensic science. During the process of drowning, diatoms enter the lungs through respiration and subsequently spread throughout the body via the bloodstream. The detection of diatoms in tissues and organs such as the liver and kidneys strongly supports the diagnosis of drowning. Additionally, identifying the species of diatoms can aid forensic science in inferring the location of the drowning.^8^^,^^9^

Currently, traditional methods for diatom detection primarily rely on observation and classification under light or electron microscopes.^10^^,^^11^ These methods are time-consuming and labor-intensive, requiring diatom taxonomists with extensive professional knowledge and experience, of whom there are very few.^12^^,^^13^ Manual identification is prone to subjective judgment by the operator, thereby reducing the accuracy of diatom classification.^14^^,^^15^ Recent developments in molecular biology have provided new methods for diatom testing.^16^^,^^17^ This method utilizes genetic markers to identify specific species. Compared to morphology-based methods, deoxyribonucleic acid (DNA) sequence-based diatom detection methods are simpler and more convenient to operate and exhibit higher sensitivity in diatom detection.^18^^,^^19^^,^^20^^,^^21^ As a result, this method has been extensively researched and applied in diatom testing. However, the samples involved in diatom research and practice are often mixtures of various diatoms, and how to achieve molecular testing of mixed diatom samples has always been a challenge in molecular species identification. The recently developed multiplex polymerase chain reaction-high-resolution melting (PCR-HRM) offers a potential solution to this problem.^22^^,^^23^ However, whether this technology can be applied to the testing of mixed diatom samples and its testing capabilities have not yet been reported.

HRM technology is an emerging genetic analysis technique.^24^ Its principle is based on the binding of double-stranded DNA with a saturated fluorescent dye. During the heating process, the double-stranded DNA gradually dissociates into single-stranded molecules and the fluorescent dye is released during this dissociation. By real-time monitoring of the changes in the fluorescent signal, a characteristic melting curve is obtained.^25^^,^^26^ HRM technology is simple to operate, and the melting reaction can be directly conducted after polymerase chain reaction (PCR) amplification, effectively reducing the risk of cross-contamination. Additionally, HRM technology features high throughput, cost-effectiveness, and broad applicability. Currently, HRM technology is widely used for species identification of microorganisms such as bacteria and viruses, but there are very few reports on the application of HRM technology in diatom testing research.^27^^,^^28^^,^^29^ HRM technology is theoretically capable of detecting all amplification products in a single tube. However, our preliminary experiments have revealed that when amplifying DNA from multiple species using a single primer, the melting curve can only form one or two characteristic peaks. This may be due to the small differences in the melting temperatures of the amplification products, resulting in merged peaks that make it difficult to effectively distinguish between species. The multiplex PCR (mPCR) technique enables the simultaneous addition of multiple pairs of specific primers targeting different regions of the same DNA template within a single PCR amplification reaction, thereby amplifying multiple target DNA fragments.^30^^,^^31^^,^^32^ We can utilize these multiple target DNA fragments amplified by mPCR and analyze them using HRM technology. By leveraging the differences in the melting temperatures of the amplified products, species identification can be achieved.

Multiplex PCR-HRM combines PCR technology with HRM curve analysis, enabling the simultaneous detection and differentiation of multiple DNA sequence variations in a sample. This allows us to analyze multiple target gene regions in a single reaction, thereby achieving species identification. This typically requires the design of multiple primer pairs to ensure that different target fragments have varying lengths or GC (guanine and cytosine) contents, thereby generating distinguishable melting curves. Therefore, this study explores the integration of HRM technology with mPCR technology to examine its value and capability in testing mixed diatom samples.

## Results

### Identification and classification of experimental diatom species

Scanning electron microscope (SEM) examination confirmed that the eight cultured diatom species exhibited morphological characteristics consistent with their purchased taxa (Figure 1).

**Figure 1:**
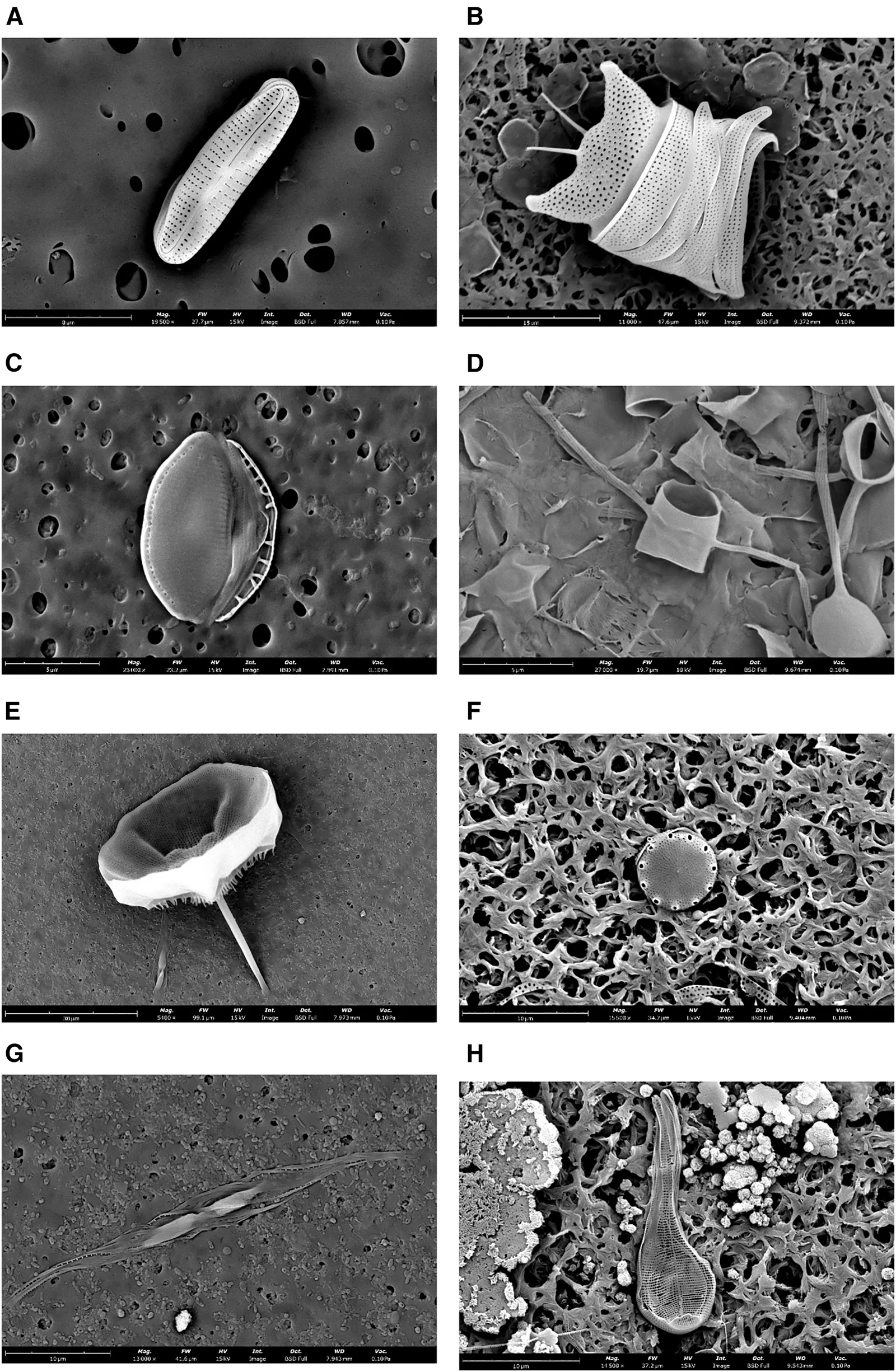
SEM examination results of 8 pure diatom species *Navicula* sp. (A), *Odontella* sp. (B), *Nitzschia* sp. (C), *Chaetoceros* sp. (D), *Ditylum* sp. (E), *Skeletonema* sp. (F), *Cylindrotheca* sp. (G), and *Asterionellopsis* sp. (H). Scale bars: 5 μm (C and D), 8 μm (A), 10 μm (F–H), 15 μm (B), and 30 μm (E), as indicated in each image.

### Results of primer design and screening

Through literature screening, we obtained a total of 18 pairs of primers, and through our own design, we acquired 65 pairs of primers. After experimental screening, 15 pairs of primers met the criteria, including 8 pairs from the literature and 7 pairs designed by ourselves (Table 1). Through repeated experiments, these primers successfully identified 1 to 8 species of the experimental diatoms.

**Table 1:** Information on diatom-specific primers

Primer	Primer sequences	Target gene	Annealing temperature (°C)	Amplicon length (bp)	Source
*UPA99-F*	GGAAACGTACAAAGGTTTCC	UPA	59	99	Liu et al.^33^
*UPA99-R*	GTCGCTGCACGACTTGTAGG				
*18S-V7F*	TCCGTTAACGAACGAGACC	18S	58	130	Zhao et al.^34^
*18S-V7R*	GGCATCACAGACCTGTTATTG				
*PsaA-2-F*	GGCACTGGATCTACACGGTA	PsaA	61	146	Vinayak et al.^35^
*PsaA-2-R*	TCCGTATCACACCACAACCA				
*rbcL197-F*	CTCAACCATTCATGCG	rbcL	47	197	Quyi Xu^36^
*rbcL197-R*	CTGTGTAACCCATAAC				
*DiatrbcL708F*	AGGTGAAGTTAAAGGTTCWTAYTTAAA	rbcL	55	312	Kezlya et al.^37^
*R3_1*	CCTTCTAATTTACCWACWACTG				
*Primer1-F*	CAATTGGAGGGCAAGTCTGG	18S	58	236	Li et al.^38^
*Primer1-R*	CCTGCACGACAACCTAATG				
*ND-F*	ATTAGGTTTATCTGGTAAAAACTAC	18S	53	113	Yu et al.^39^
*ND-R*	TTCTCTCCAACGCATGAA				
*DNGT-F*	CCGTTCTTGACTGGAATCGT	LSU	60	254	Yanjie Sun^40^
*DNGT-R*	AAACCACAAGGGGCTTAGGA				
*18S-333NF*	GTGGCATTCAGTTGTCGTG	18S	58	333	self-designed
*18S-333NR*	CCCTTGTCGGCATAGTTTA				
*18S-341JF*	CGTTCTGGCATTAGGTTGT	18S	55	341	
*18S-341JR*	ATCCTGAGTCGGCATAGTT				
*rbcL-256ZF*	CATCGTGCTGGTAACTCT	rbcL	60	256	self-designed
*rbcL-256ZR*	ACGCAACAGGCATACATT				
*18S-196OF*	CGGAGGTGCTTTGGTGAT	18S	60	196	
*18S-196OR*	CTGCCTTCCTTGGATGTG				
*rbcL-NF*	AAACATTCCAAGGTCCAG	rbcL	55	419	
*rbcL-NR*	TTCACGAGCCCATAAAG				
*18S-356HF*	GGGATACCCATCGTTTACT	18S	58	356	
*18S-356HR*	GATTTCTCATACGGTGCTG				
*28S-191CF*	ACGGACCAAGGAGTCTAAC	28S	58	191	
*28S-191CR*	CTGGCTTCACCCTATTCA				

### Single-primer HRM curves of single diatoms distinguish diatom species

After PCR amplification of a single diatom using a single primer pair, HRM analysis was performed, with the experimental results shown in Figure 2. All of the following melting curve plots are derivative melting curves obtained from HRM analysis. When employing HRM analysis, each amplification product forms a distinct characteristic peak. The horizontal coordinate corresponding to the highest point of the characteristic peak represents the melting temperature. Different amplification products yield characteristic peaks with varying shapes at distinct positions, enabling the identification of different diatom species based on this feature. The primers *UPA99*, *18S-V7*, *PsaA-2*, *rbcL197*, *DiatrbcL708*, *Primer1*, *ND*, *28S-191C*, and *18S-333N* were all capable of specifically amplifying eight species of marine diatoms, and each primer yielded eight distinct melting curves following HRM analysis. The primers *18S-356H* and *18S-341J* were able to specifically amplify seven species of marine diatoms, resulting in seven different melting curves after HRM analysis. The primer *rbcL-256Z* specifically amplified four species of marine diatoms, producing four distinct melting curves upon HRM analysis. The *18S-196O* and *rbcL-N* primers were able to specifically amplify only two species of marine diatoms, and only two distinct melting curves were obtained after HRM analysis. The *DNGT* primer were able to specifically amplify *Skeletonema* sp. No detection peaks were observed in all negative control groups. These results indicate that HRM technology can differentiate between different diatom species, with varying numbers of diatoms amplified by different primers and different melting temperatures were obtained (Table 2).

**Figure 2:**
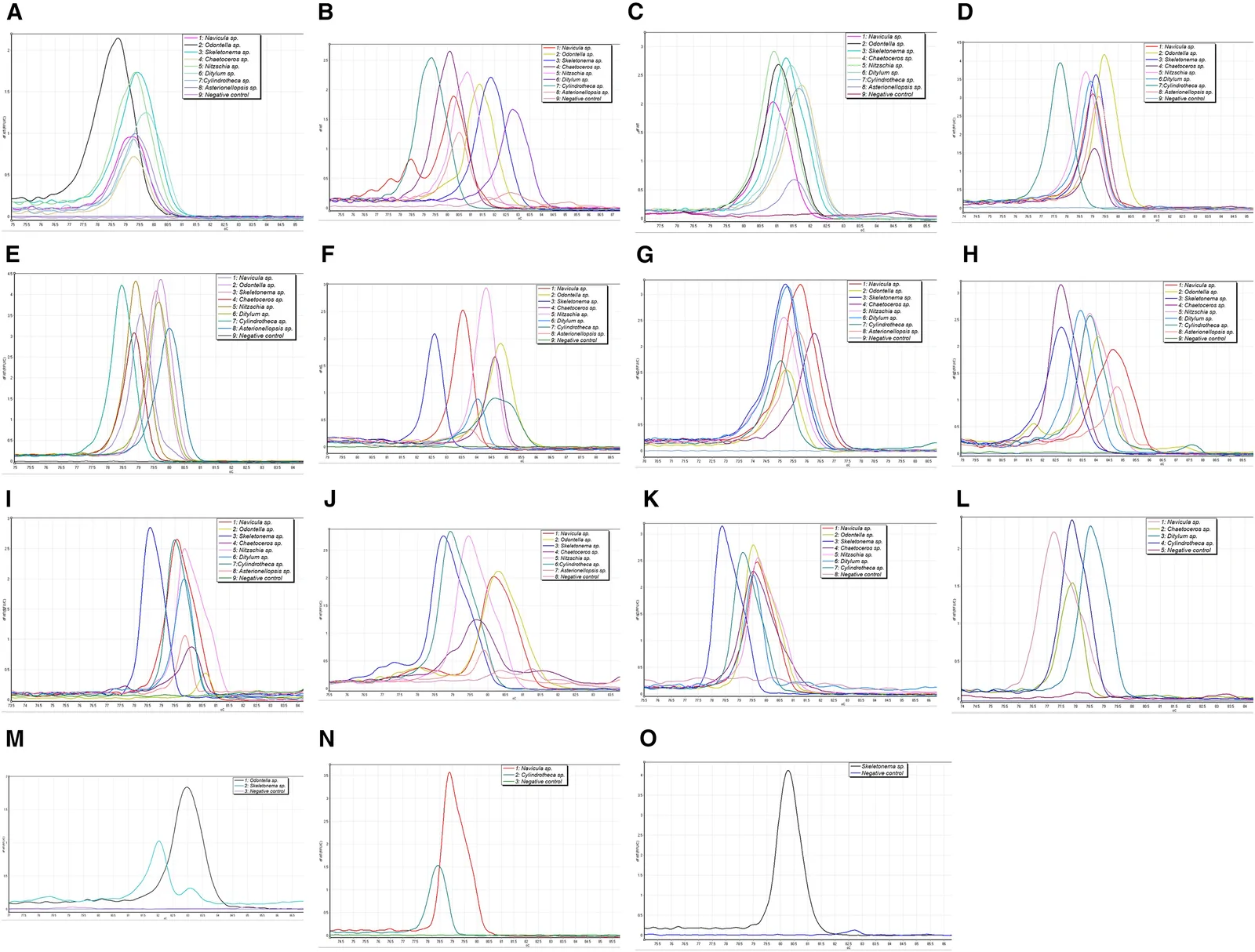
Different primers yield specific HRM curves for diatoms (A) Primer *UPA99* could specifically amplify 8 species of marine diatoms. (B) Primer *18S-V7* could specifically amplify 8 species of marine diatoms. (C) Primer *PsaA-2* could specifically amplify 8 species of marine diatoms. (D) Primer *rbcL197* could specifically amplify 8 species of marine diatoms. (E) Primer *DiatrbcL708* could specifically amplify 8 species of marine diatoms. (F) Primer *Primer1* could specifically amplify 8 species of marine diatoms. (G) Primer *ND* could specifically amplify 8 species of marine diatoms. (H) Primer *28S-191C* could specifically amplify 8 species of marine diatoms. (I) Primer *18S-333N* could specifically amplify 8 species of marine diatoms. (J) Primer *18S-356H* could specifically amplify 7 species of marine diatoms. (K) Primer *18S-341J* could specifically amplify 7 species of marine diatoms. (L) Primer *rbcL-256Z* could specifically amplify 4 species of marine diatoms. (M) Primer *18S-196O* could only specifically amplify two species of marine diatoms. (N) Primer *rbcL-N* could only specifically amplify two species of marine diatoms. (O) Primer *DNGT* could specifically amplify *Skeletonema* sp. only. Representative results from 3 independent experiments (*n* = 3) are shown. See also Figures S1 and S2.

**Table 2:** Melting temperatures of different diatoms amplified by different primers (°C)

Primer	*Navicula* sp.	*Odontella* sp.	*Skeletonema* sp.	*Chaetoceros* sp.	*Nitzschia* sp.	*Ditylum* sp.	*Cylindrotheca* sp.	*Asterionellopsis* sp.
*UPA99*	79.25	78.73	79.40	79.28	79.35	79.72	79.28	79.35
*18S-V7*	80.27	81.37	81.85	80.10	80.85	82.77	79.32	80.50
*PsaA-2*	80.88	81.05	81.28	81.75	80.90	81.42	81.65	81.52
*rbcL197*	79.00	79.45	79.13	79.08	78.75	78.93	77.75	79.25
*DiatrbcL708*	79.08	79.72	79.57	78.85	78.90	79.65	78.45	80.00
*Primer1*	83.55	84.80	82.58	84.62	84.32	84.03	84.60	83.80
*ND*	75.75	75.23	75.20	76.28	75.15	75.28	75.03	75.65
*DNGT*	–	–	80.28	–	–	–	–	–
*18S-333N*	79.57	80.62	78.60	80.10	79.85	79.85	79.48	79.87
*18S-341J*	79.67	79.53	78.37	79.52	79.70	79.53	–	79.15
*rbcL-256Z*	77.25	–	–	77.90	–	78.53	77.87	–
*rbcL-N*	78.90	–	–	–	–	–	78.43	–
*18S-356H*	80.18	80.30	78.75	79.68	79.45	–	78.95	79.90
*28S-191C*	84.63	84.08	82.70	82.70	83.75	83.42	83.78	84.80
*18S-196O*	–	82.97	82.03	–	–	–	–	–

“–” indicates that the primer does not amplify the corresponding algal species.

### HRM analysis cannot readily distinguish single-primer-amplified pairwise diatom mixtures

We performed HRM analysis on mixed diatom samples using the primers *DiatrbcL708* and *28S-191C*, where the mixed samples consisted of pairwise combinations of diatoms. The results in Figure 3A show that, except for the mixture of *Navicula* sp. and *Asterionellopsis* sp., which exhibited the characteristic peak of *Navicula* sp., the pairwise mixtures with the other six diatom species only displayed the characteristic peaks of the respective other diatoms, without forming double peaks with *Navicula* sp. As shown in Figure 4A, when *Navicula* sp. is mixed with *Chaetoceros* sp., *Nitzschia* sp., or *Cylindrotheca* sp. it can form a double peak, while the rest display the characteristic peak of *Navicula* sp. As shown in Figures 3B–3G and Figures 4B–4G, the formation of 2 to 3 double peaks can be observed, while the rest remain single peaks. The results in Figure 3H show that when the other seven species of diatoms were mixed with *Asterionellopsis* sp., only the characteristic peaks of the other diatoms were displayed, and no double peaks were formed with *Asterionellopsis* sp. As shown in Figure 4H, except for forming double peaks with *Skeletonema* sp., *Asterionellopsis* sp. displayed the characteristic peaks of the remaining six species of diatoms when mixed with them. From these results, it can be observed that these eight species of diatoms exhibit suppression or characteristic peak fusion in different mixtures, especially *Asterionellopsis* sp., which is highly susceptible to suppression in mixed systems.

**Figure 3:**
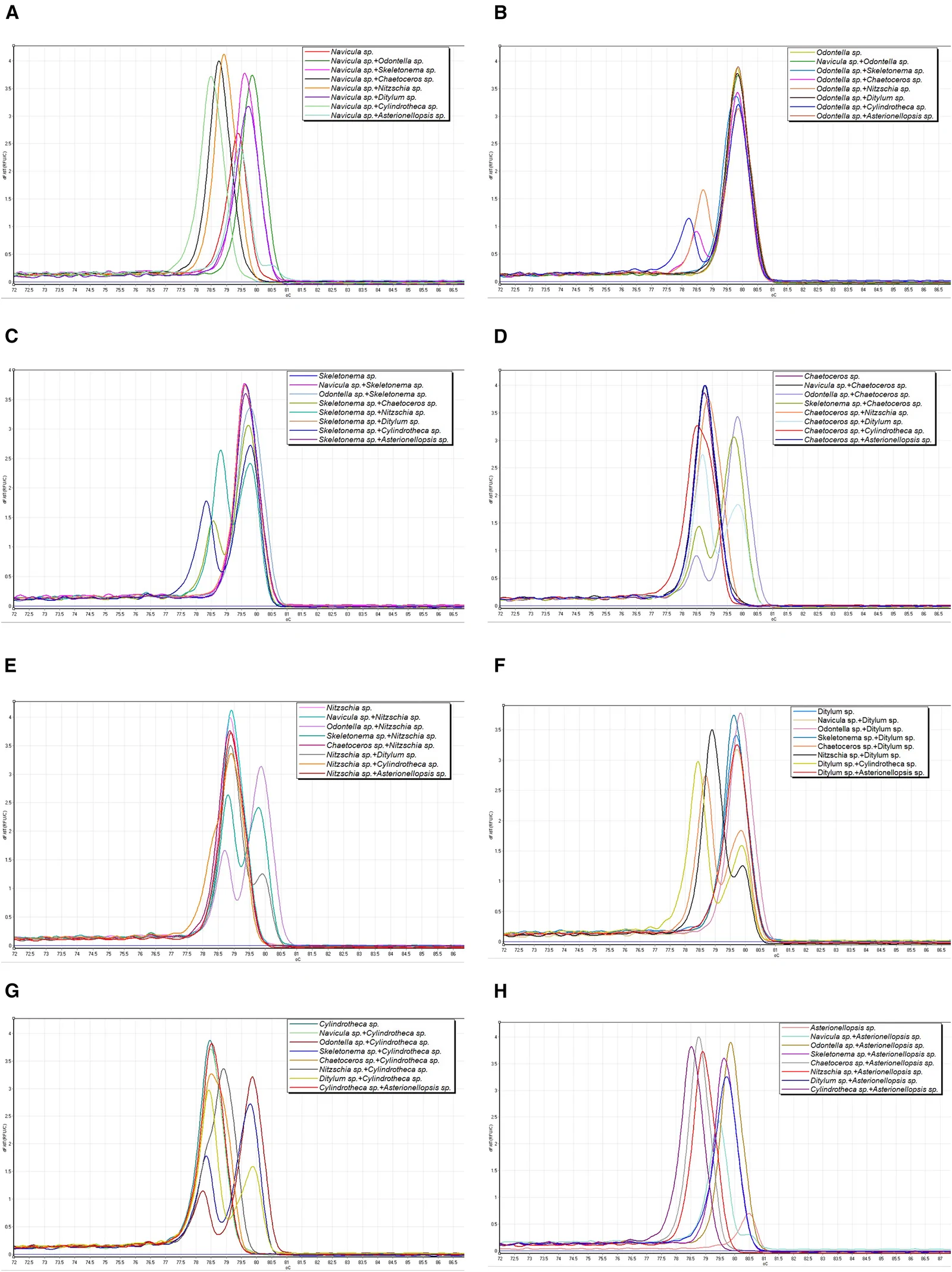
HRM analysis using primer *DiatrbcL708* The melting curves obtained from pairwise mixtures of *Navicula* sp. (A), *Odontella* sp. (B), *Skeletonema* sp. (C), *Chaetoceros* sp. (D), *Nitzschia* sp. (E), *Ditylum* sp. (F), *Cylindrotheca* sp. (G), and *Asterionellopsis* sp. (H), each mixed with the other seven diatom species (excluding itself). Representative results from 3 independent experiments (*n* = 3) are shown. See also Figure S3.

**Figure 4:**
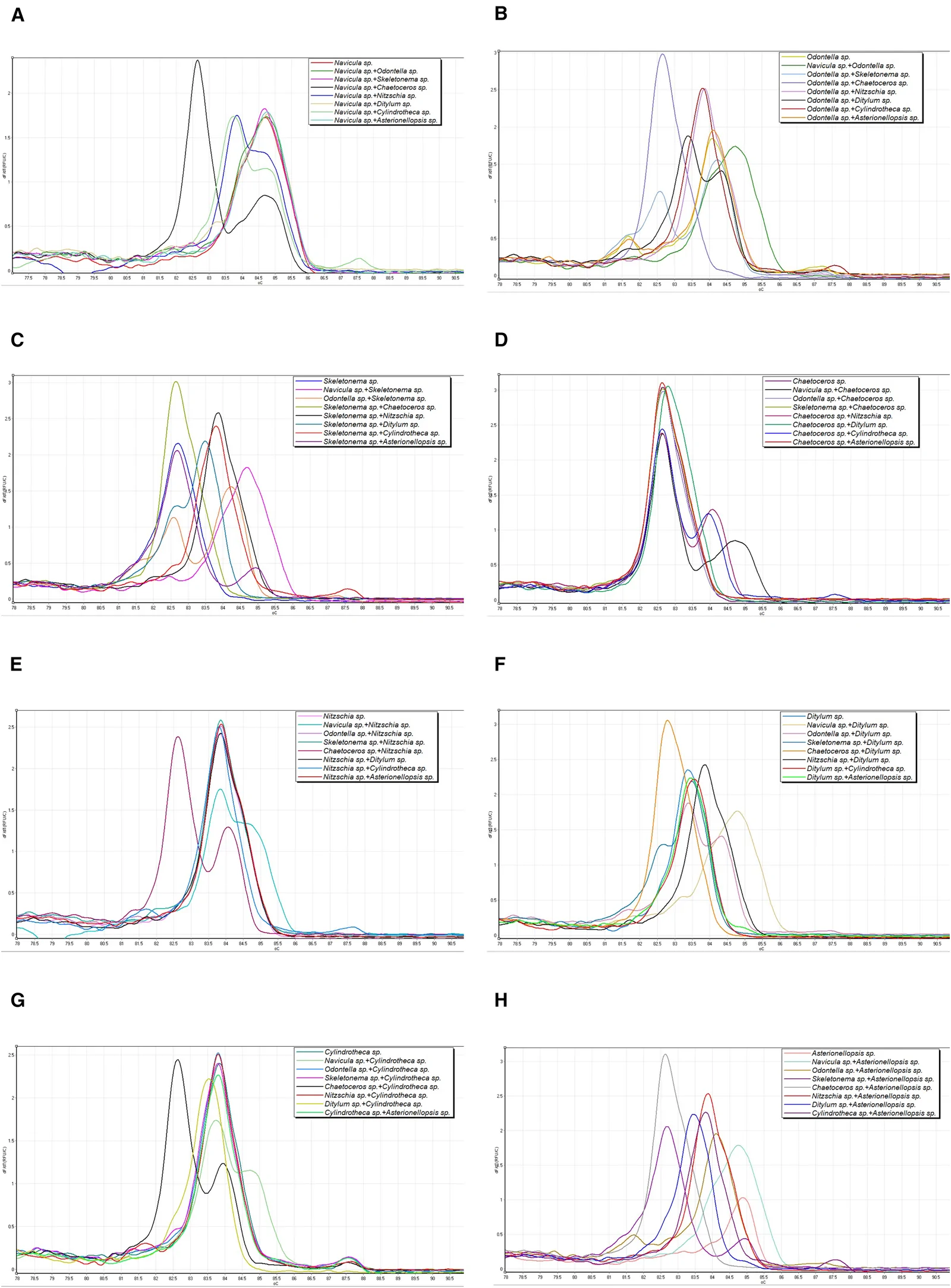
HRM analysis using primer *28S-191C* The melting curves obtained from pairwise mixtures of *Navicula* sp. (A), *Odontella* sp. (B), *Skeletonema* sp. (C), *Chaetoceros* sp. (D), *Nitzschia* sp. (E), *Ditylum* sp. (F), *Cylindrotheca* sp. (G), and *Asterionellopsis* sp. (H), each mixed with the other seven diatom species (excluding itself). Representative results from 3 independent experiments (*n* = 3) are shown. See also Figure S4.

### Single-primer HRM of an eight-diatom mixture cannot accurately identify all species

The PCR amplification of mixed samples of eight diatom species was conducted using primers *DiatrbcL708* and *28S-191C*, followed by HRM analysis. The results showed that the amplification of mixed diatom samples with both the primers only formed double peaks, which were unable to accurately identify the species of the eight diatoms in the mixed samples (Figure 5). Similarly, the double peaks formed by the mixed samples could not accurately correspond to which two diatom species they represented. This indicates that when using a single primer for HRM analysis of mixed diatoms, the detection capability is reduced and accurate species identification of the eight diatoms cannot be achieved.

**Figure 5:**
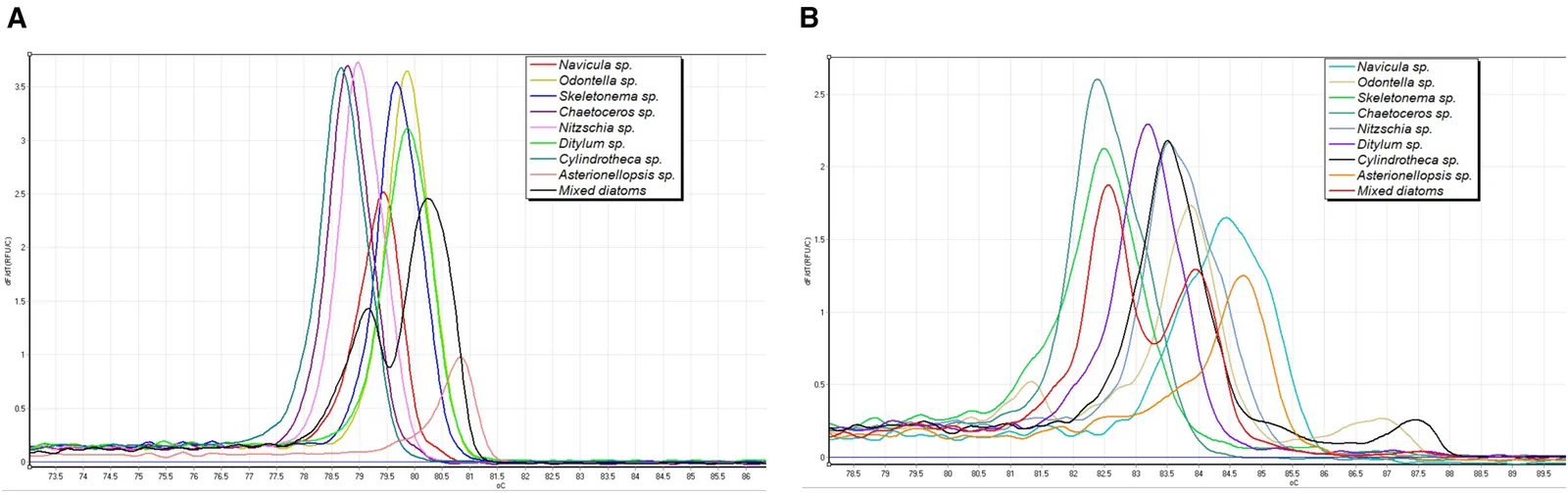
HRM with primers *DiatrbcL708* and *28S-191C* for eight mixed diatom species, respectively (A) Melting curve of eight mixed diatom samples amplified with primer *DiatrbcL708*. (B) Melting curves of eight mixed diatom samples amplified with primer *28S-191C*. The curve labeled “mixed diatoms” represents the mixed sample, while the others are positive controls. Representative results from 3 independent experiments (*n* = 3) are shown. See also Figure S5.

### Multiple primer mixture combined with HRM enables rapid identification of diatom species

We used a mixture of two primers, *28S-191C* and *PsaA-2*, with similar annealing temperatures to amplify a single diatom in one reaction tube. The results showed that the mixture of the two primers could form characteristic double peaks when amplifying the diatom (Figure 6). Each peak corresponded to the characteristic peak formed by the amplification of the same diatom with the respective primer, with no occurrence of amplification inhibition or fusion of characteristic peaks. This indicates that multiple primers can be mixed for rapid species identification of a single diatom. We also employed other primer combinations for multiplex PCR-HRM analysis of single diatoms (Figure 7). From these experimental results, it is evident that using a mixture of two primers allows rapid and accurate identification of diatom species.

**Figure 6:**
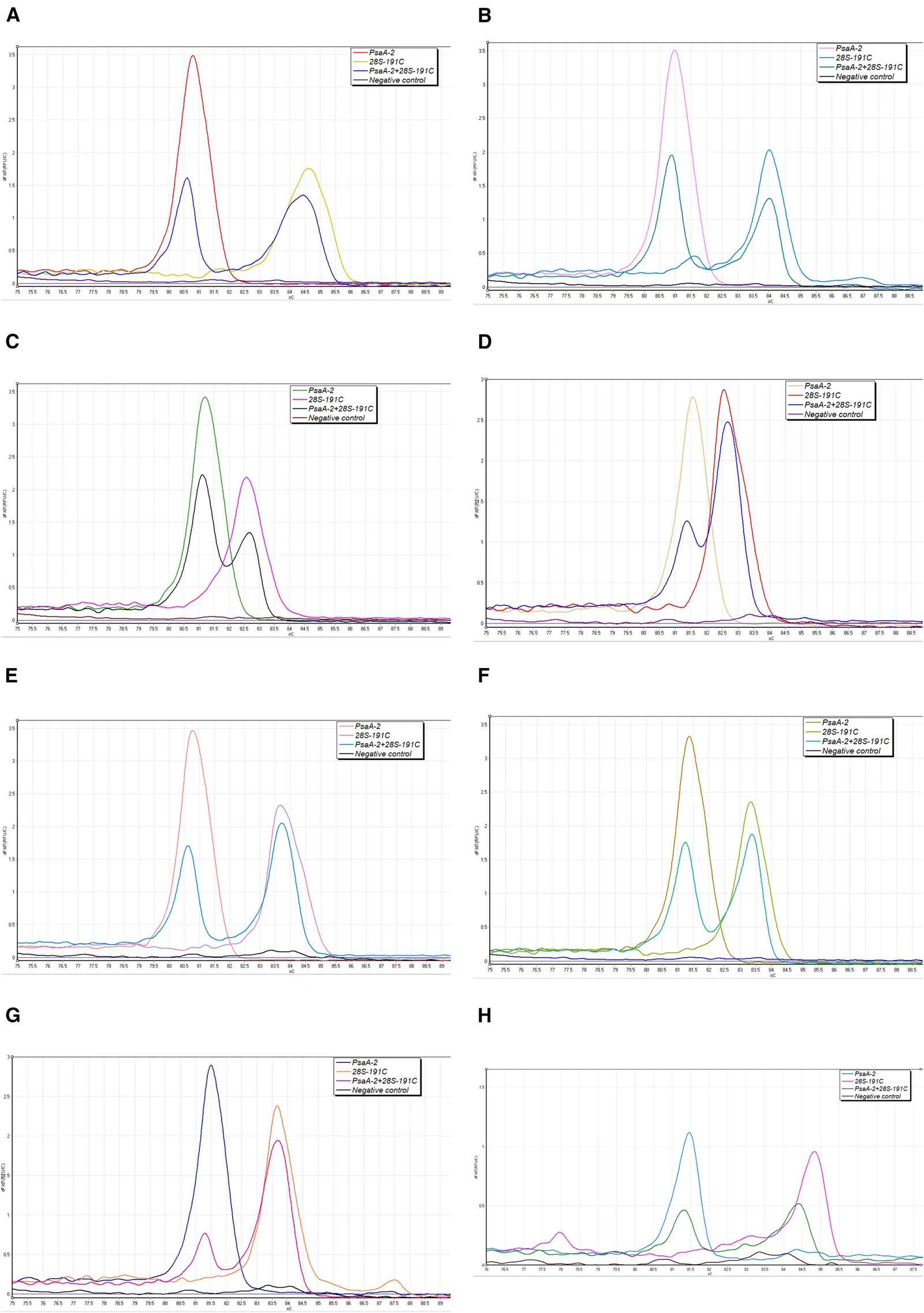
HRM with mixed primers *28S-191C* and *PsaA-2* rapidly identifies eight diatom species *Navicula* sp. (A), *Odontella* sp. (B), *Skeletonema* sp. (C), *Chaetoceros* sp. (D), *Nitzschia* sp. (E), *Ditylum* sp. (F), *Cylindrotheca* sp. (G), and *Asterionellopsis* sp. (H). Representative results from 3 independent experiments (*n* = 3) are shown. See also Figure S6.

**Figure 7:**
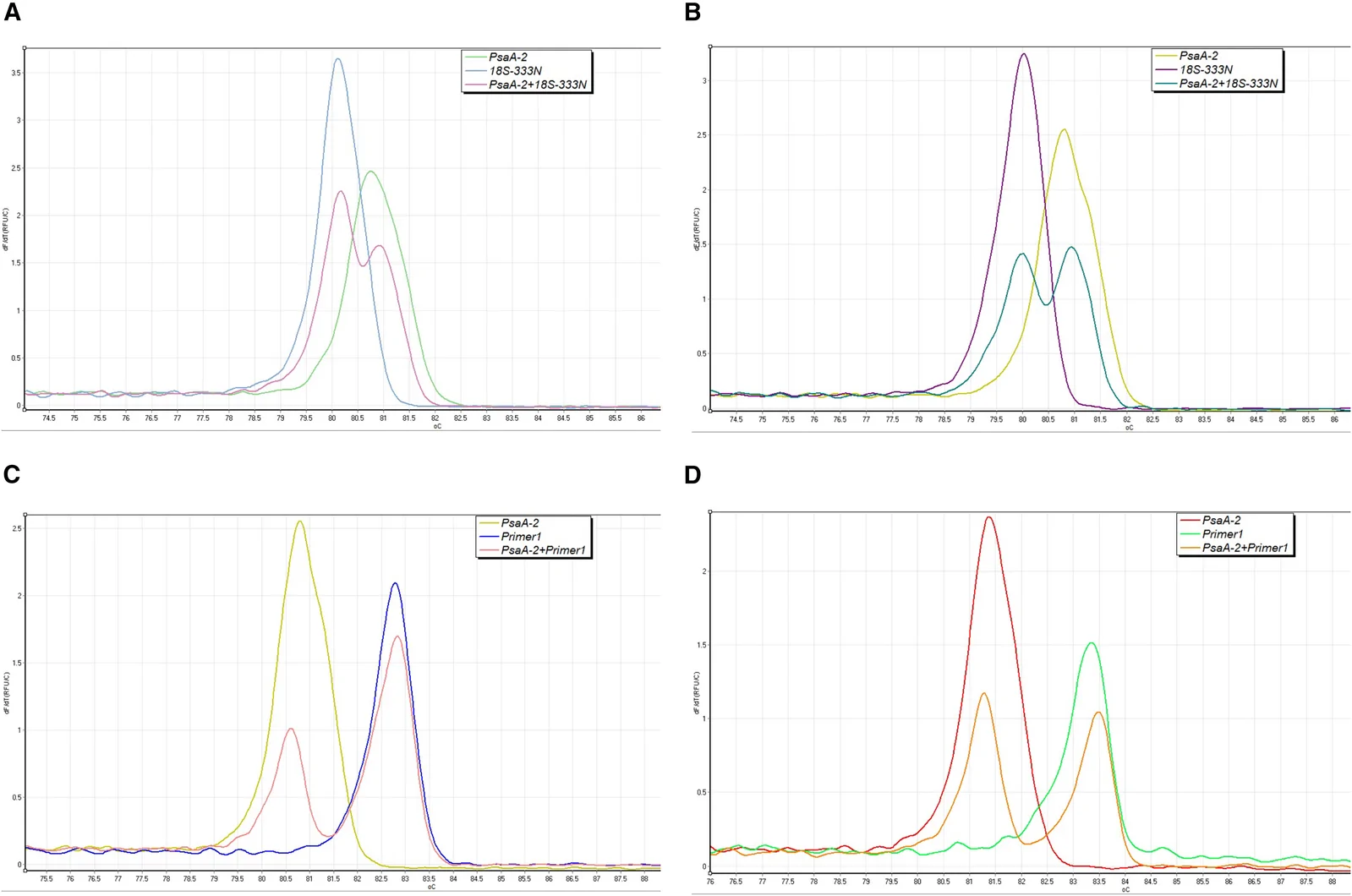
HRM with different primer combinations identifies single diatom species (A and B) The melting curves obtained by mixed amplification of *Nitzschia* sp. and *Navicula* sp. using primers *PsaA-2* and *18S-333N*, respectively. (C and D) The melting curves obtained by mixed amplification of *Navicula* sp. and *Ditylum* sp. using primers *PsaA-2* and *Primer1*, respectively. Representative results from 3 independent experiments (*n* = 3) are shown. See also Figure S7.

### Multiple primer pairs with HRM identify mixed diatom species

Due to the decreased ability of a single primer to discriminate among mixed diatom samples, it is not possible to accurately identify the species of diatoms in mixed diatom samples. Therefore, we applied multiple primer pairs for the identification of diatom species. Through our research, we found that using a combination of the *18S-196O*, *rbcL-N*, and *DNGT* primers enables the differentiation of four diatom species in mixed samples. The results showed that the multiplex PCR-HRM reaction system could distinguish four diatom species: *Odontella* sp., *Navicula* sp., *Cylindrotheca* sp., and *Skeletonema* sp. The shape and position of the melting curves formed after amplification of the mixed samples of the four diatom species and the mixed samples of the eight diatom species are almost identical, and the characteristic peaks formed by single samples and mixed samples are closely located (Figure 8). Therefore, this amplification system can distinguish between *Odontella* sp., *Navicula* sp., *Cylindrotheca* sp., and *Skeletonema* sp., while simultaneously demonstrating the presence of these four diatom species in mixed samples. Thus, we have preliminarily established a multiplex PCR-HRM system for the identification of mixed diatom samples.

**Figure 8:**
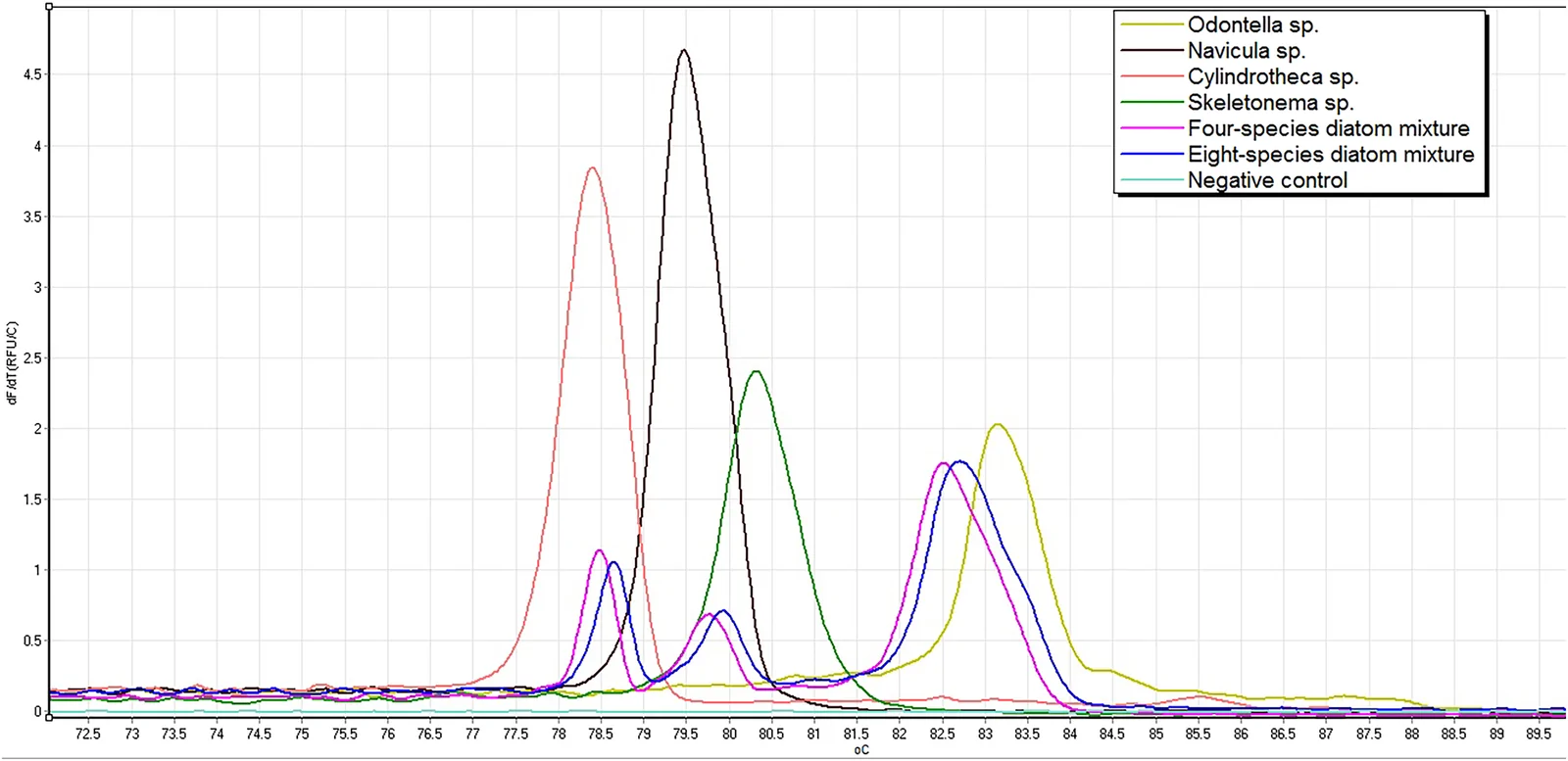
Melting curve analysis of multiplex PCR-HRM amplification products *Odontella* sp., *Navicula* sp., *Skeletonema* sp., and *Cylindrotheca* sp., a mixture of these four diatom species, and a mixture of eight diatom species were amplified using primers *18S-196O*, *rbcL-N*, and *DNGT*. Representative results from 3 independent experiments (*n* = 3) are shown. See also Figure S8.

## Discussion

This study used eight pure strains of diatoms as research objects. From 83 primer pairs designed internally or selected from the literature, 15 primer pairs capable of diatom detection were validated through experiments. HRM analysis following PCR amplification of single diatom samples with a single primer pair enabled rapid identification of diatom species. This demonstrates the application value of HRM technology for the detection of individual diatom species. The melting temperatures of different diatoms amplified by various primer pairs obtained in this study provide experimental data references for establishing an HRM technical system for identifying seawater diatom species in related research.

However, in natural water environments, mixed diatom samples are often encountered. Among the molecular species identification techniques for mixed species samples, high-throughput sequencing technology is currently a more effective approach.^19^^,^^41^ It can simultaneously obtain DNA sequence data from a large number of species, significantly improving the efficiency of species identification. When dealing with complex, mixed, or unknown samples, this technology has the potential to reveal species information within them or even discover unknown species. Despite the advantages of high-throughput sequencing technology, its application is limited by factors such as high costs, long experimental cycles, and the vast and complex data analysis. Additionally, it heavily relies on the completeness and reliability of reference databases.^42^ These factors collectively constrain the widespread use of this technology. Although multiplex PCR-HRM technology cannot replace high-throughput sequencing technology, it also has certain advantages in mixed species identification. Multiplex PCR-HRM technology completes the PCR amplification and HRM detection process in the same closed tube without any intermediate operations. This not only reduces the risk of cross-contamination but also simplifies the process and saves time. This technology only requires a high-resolution real-time fluorescence PCR instrument and some corresponding reagents, resulting in relatively low costs.

During the analysis of pairwise mixed diatom samples and a mixture of eight diatom species, we observed mutual inhibition during amplification or fusion of similar characteristic peaks among diatoms. This indicates that a single primer pair has reduced species identification capability for mixed diatom samples and cannot accurately determine the species composition of diatoms in such samples. Therefore, we attempted to combine HRM technology with mPCR to identify diatom species in mixed samples. By combining different primers, we initially constructed a multiplex PCR-HRM system for the identification of mixed diatom samples.

In constructing the multiplex PCR-HRM system, the design and selection of primers are the key steps, directly determining success or failure. The annealing temperatures of primers should not differ too much. However, the melting temperatures of the amplified products must not be too close; if the melting temperatures are similar, fusion peaks will form, making it impossible to distinguish species. Studies have shown that in HRM analysis, when the length of the amplified product is too long (400–1,000 bp), its sensitivity and specificity will slightly decrease (by 3.9% and 0.6%, respectively).^43^^,^^44^ The most common problem in this experiment is complementarity between primers, resulting in excessive primer dimers. However, during HRM analysis, the presence of primer dimers can be observed intuitively, and there is no need to use agarose gel electrophoresis for observation.

The amplification results of the multiplex PCR-HRM system are also influenced by some other factors, such as the ratio of primers, the concentration of dNTPs, and the concentration of Mg^2+^. Studies have shown that excessively high primer concentrations can lead to competition among primers for the materials required for PCR, thereby causing amplification imbalance at different loci and resulting in experimental failure.^45^^,^^46^ dNTPs are the raw materials for synthesizing DNA strands in PCR. When the concentration of dNTPs is insufficient, the yield of amplification products decreases. In mPCR, some primers may fail to amplify due to a lack of raw materials. Mg^2+^ is an essential cofactor for DNA polymerase function. An insufficient concentration of Mg^2+^ can affect the activity of the polymerase, thereby reducing PCR amplification efficiency. Although an excessively high Mg^2+^ concentration increases the stability between primers and templates, it also increases nonspecific amplification.^45^^,^^47^^,^^48^

When we performed HRM analysis on eight different diatom species using a single primer, we found that the melting temperatures of the amplification products obtained by amplifying different diatoms with the same primer were sometimes identical or differed by less than 0.1°C. For example, the melting temperature of both *Chaetoceros* sp. and *Cylindrotheca* sp. amplified by primer UPA99 was 79.28°C. This could be due to the similarity of the two amplified sequences or it could be caused by fluctuations in temperature differences between wells leading to identical melting temperatures. Therefore, we addressed this issue by mixing two primers in the same reaction tube to amplify the diatoms. If the combination of two primers still cannot identify the species, a combination of three primers can be used, but the more primers used, the more difficult the experiment becomes.

### Limitations of the study

The reaction system constructed in this study can currently detect only four types of diatoms from a mixture of eight samples. This may be related to the insufficient number of specific primers and the differences in amplification efficiency among these primers. Therefore, we will continue to design different primers for various diatoms to construct a multiplex PCR-HRM system capable of detecting more diatoms. At the same time, we will optimize the multiplex PCR-HRM system by adjusting the PCR reaction system, verifying the sensitivity of each primer, and adjusting the concentration ratio between primers. This study did not validate the system using water samples from the environment. Therefore, the performance of this method in unknown water samples remains unclear. In the future, we will test water samples from the environment to evaluate their practical application value in environmental detection and forensic practice. Nevertheless, this study preliminarily demonstrates the feasibility of the multiplex PCR-HRM system for analyzing mixed diatom samples. Moreover, this method enables rapid detection of diatoms, with high specificity, low cost, and simple operation. It can be implemented in basic laboratories and shows promising application prospects.

## Resource availability

### Lead contact

Requests for further information, resources, and data should be directed to and will be fulfilled by the lead contact, Yunchao Zhou (hyzhouyc@163.com).

### Materials availability

This study did not generate new unique reagents.

### Data and code availability

• All data reported in this paper will be shared by the lead contact upon request.
• This paper does not report original code.
• Any additional information required to reanalyze the data reported in this paper is available from the lead contact upon request.

## Acknowledgments

This research was funded by the 10.13039/501100001809National Natural Science Foundation of China (grant nos. 82060341 and 81560304), the Academician Innovation Platform Scientific Research Project of Hainan Province (grant no. YSPTZX202134), and the Innovation for Graduate Students in Hainan province (grant no. Qhys2023-474).

## Author contributions

Y.Z., B.C., J.C., and J.D. conceptualized the study and designed the methodology. Y.Z. and Y.L. contributed to the bench experiments and data collection. Y.Z. and Y.L. performed data analysis and visualization. Y.Z., B.C., J.C., and J.D. provided supervision for the project. Y.Z. and J.D. drafted the manuscript. Y.Z., B.C., J.C., and J.D. reviewed and edited the manuscript.

## Declaration of interests

The authors declare no competing interests.

## Declaration of generative AI and AI-assisted technologies in the manuscript preparation process

During the preparation of this manuscript, the authors used DeepSeek, developed by Hangzhou DeepSeek Artificial Intelligence Co., Ltd., to perform language translation of parts of the manuscript. After using this tool, the authors reviewed and revised the content as necessary and take full responsibility for the content of the published work.

## STAR★Methods

### Key resources table

**Table:** 

REAGENT or RESOURCE	SOURCE	IDENTIFIER
**Chemicals, peptides, and recombinant proteins**		

Eva Green Fluorescent Dye	Biotium	31000

**Critical commercial assays**		

Pfu PCR Mix	TIANGEN BIOTECH (BEIJING) Co., LTD.	1109499
Enriched Seawater Medium “f/2”	the Seaweed Culture Collection Center, Institute of Oceanology, Chinese Academy of Sciences	N/A
Erdschreiber	the Freshwater Algae Culture Collection at the Institute of Hydrobiology	N/A

**Experimental models: Organisms/strains**		

*Nitzschia sp.*	the Seaweed Culture Collection Center, Institute of Oceanology, Chinese Academy of Sciences	MASCC-0037
*Odontella sp.*	the Seaweed Culture Collection Center, Institute of Oceanology, Chinese Academy of Sciences	MASCC-0019
*Chaetoceros sp.*	the Seaweed Culture Collection Center, Institute of Oceanology, Chinese Academy of Sciences	MASCC-0031
*Navicula sp.*	the Seaweed Culture Collection Center, Institute of Oceanology, Chinese Academy of Sciences	MASCC-0035
*Skeletonema sp.*	the Seaweed Culture Collection Center, Institute of Oceanology, Chinese Academy of Sciences	MASCC-0022
*Cylindrotheca sp.*	the Freshwater Algae Culture Collection at the Institute of Hydrobiology	FACHB-2185
*Ditylum sp.*	the Freshwater Algae Culture Collection at the Institute of Hydrobiology	FACHB-2559
*Asterionellopsis sp.*	the Freshwater Algae Culture Collection at the Institute of Hydrobiology	FACHB-2578

**Oligonucleotides**		

*UPA99-F:* GGAAACGTACAAAGGTTTCC	Liu et al.^33^	N/A
*UPA99-R: GTCGCTGCACGACTTGTAGG*	Liu et al.^33^	N/A
*18S-V7F: TCCGTTAACGAACGAGACC*	Zhao et al.^34^	N/A
*18S-V7R: GGCATCACAGACCTGTTATTG*	Zhao et al.^34^	N/A
*PsaA-2-F: GGCACTGGATCTACACGGTA*	Vinayak^35^	N/A
*PsaA-2-R: TCCGTATCACACCACAACCA*	Vinayak^35^	N/A
*rbcL197-F: CTCAACCATTCATGCG*	Xu^36^	N/A
*rbcL197-R: CTGTGTAACCCATAAC*	Xu^36^	N/A
*DiatrbcL708F: AGGTGAAGTTAAAGGTTCWTAYTTAAA*	Kezlya et al.^37^	N/A
*R3_1: CCTTCTAATTTACCWACWACTG*	Kezlya et al.^37^	N/A
*Primer1-F: CAATTGGAGGGCAAGTCTGG*	Li et al.^38^	N/A
*Primer1-R: CCTGCACGACAACCTAATG*	Li et al.^38^	N/A
*ND-F: ATTAGGTTTATCTGGTAAAAACTAC*	Yu et al.^39^	N/A
*ND-R: TTCTCTCCAACGCATGAA*	Yu et al.^39^	N/A
*DNGT-F: CCGTTCTTGACTGGAATCGT*	Sun^40^	N/A
*DNGT-R: AAACCACAAGGGGCTTAGGA*	Sun^40^	N/A
*18S-333NF: GTGGCATTCAGTTGTCGTG*	This study	N/A
*18S-333NR: CCCTTGTCGGCATAGTTTA*	This study	N/A
*18S-341JF* *CGTTCTGGCATTAGGTTGT*	This study	N/A
*18S-341JR: ATCCTGAGTCGGCATAGTT*	This study	N/A
*rbcL-256ZF: CATCGTGCTGGTAACTCT*	This study	N/A
*rbcL-256ZR: ACGCAACAGGCATACATT*	This study	N/A
*18S-196OF: CGGAGGTGCTTTGGTGAT*	This study	N/A
*18S-196OR: CTGCCTTCCTTGGATGTG*	This study	N/A
*rbcL-NF: AAACATTCCAAGGTCCAG*	This study	N/A
*rbcL-NR: TTCACGAGCCCATAAAG*	This study	N/A
*18S-356HF: GGGATACCCATCGTTTACT*	This study	N/A
*18S-356HR: GATTTCTCATACGGTGCTG*	This study	N/A
*28S-191CF: ACGGACCAAGGAGTCTAAC*	This study	N/A
*28S-191CR: CTGGCTTCACCCTATTCA*	This study	N/A

**Software and algorithms**		

Primer Premier 5.0	Premier	N/A
ClustalX 1.83	Higgins D.G	N/A

**Other**		

Real-time PCR cycler and High Resolution Melt analyzer	QIAGEN	Rotor-Gene Q

### Experimental model and study participant details

#### Diatoms

This study selected eight species of pure marine diatoms as the subjects. Strains including *Nitzschia sp.*, *Odontella sp.*, *Skeletonema sp.*, *Chaetoceros sp.*, and *Navicula sp.* were purchased from the Seaweed Culture Collection Center, Institute of Oceanology, Chinese Academy of Sciences, and were cultured in Enriched Seawater Medium “f/2”. Strains including *Cylindrotheca sp.*, *Ditylum sp.*, and *Asterionellopsis sp.* were purchased from the Freshwater Algae Culture Collection at the Institute of Hydrobiology, and were cultured in Erdschreiber medium. Cultivation conditions: temperature 21°C, light intensity 1500 lux, photoperiod set to 12 hours light/12 hours dark. The diatom used in the above experiment was confirmed to be of the correct diatom genus by electron microscopy prior to the experiment.

### Method details

#### DNA extraction from diatom

Diatom cells possess a unique structure with a hard silica shell. This study employs a diatom DNA extraction method that was designed and improved by our laboratory in previous research, with specific details provided in reference.^49^

#### Primer screening and design

Literature Screening: Identifying currently available diatom primer sequences that have been validated through research by conducting a literature search.

Self-designed: Retrieve the 18S, 28S, and ITS2 gene sequences of the ribosome, the COI gene sequence of the mitochondria, and the rbcL, PsaA, and PsbA gene sequences of the chloroplast from eight species of diatoms in the NCBI database (accession number in Table S1). Download the complete series in FASTA format and perform multiple sequence alignment analysis using ClustalX 1.83 to identify specific regions of different algal genera. Apply the software Primer Premier 5.0 for the design of diatom-specific primers. When designing primers, in addition to considering the basic principles of primer design, the principles of HRM technology and multiplex PCR technology should also be taken into account. The annealing temperatures of primers should differ by less than 4°C; otherwise, they cannot react in the same PCR system. The primers designed for use were synthesized by Taihe Biotechnology (Beijing) Co., Ltd.

#### Primer verification

Gradient PCR amplification and HRM analysis were performed using both searched and self-designed primers. After gradient PCR amplification, HRM analysis was conducted to determine the optimal annealing temperature for different primers and the melting temperature of the amplified products from each primer amplifying different diatoms. The PCR reaction system consisted of a 20 μL volume: 2.0 μL DNA template, 1.0 μL each of 10 μM specific forward and reverse primers, 10.0 μL of 2× Pfu PCR Mix, 1.0 μL of 20× Eva Green fluorescent dye, and 5.0 μL ddH_2_O. The PCR reaction conditions were as follows: initial denaturation at 95°C for 5 min; followed by 35 cycles of denaturation at 94°C for 30 s, annealing at 58°C (see Table 1 for details) for 30 s, and extension at 72°C for 1 min; and a final extension at 72°C for 5 min. Subsequently, HRM analysis was performed: the melting temperature was gradually increased from 60°C to 95°C at a rate of 0.1 °C/s. After the above experiments, samples showing stable and distinguishable HRM typing curves were considered to have passed validation.

#### Establishment of multiplex PCR-HRM reaction system

After confirming the effectiveness of each specific primer, a preliminary multiplex PCR-HRM reaction system was established. Since water samples often contain mixed diatom samples, this testing system is conducted in three steps. First, a single primer is used to test a mixed sample of various diatom species to observe the testing capability of a single primer on mixed diatoms. Subsequently, pairwise mixed samples of diatoms were analyzed to detect whether there is mutual inhibition during amplification or fusion of characteristic peaks due to similar melting temperatures among different diatom species, thereby screening for mixed primer testing. Finally, multiple pairs of primers were mixed for the identification of diatom species. The mixed samples and primers were combined in a 1:1 ratio (with DNA concentrations around 10 ng/μL). Meanwhile, when using a single primer to amplify a single diatom, some primers may exhibit the same or very similar melting temperatures when amplifying different diatoms, making it impossible to distinguish which species it is when identifying unknown samples. Therefore, we use multiple primers for HRM analysis to better identify the species.

The reaction system includes: 10 μL of 2×Pfu PCR Mix, 1 μL each of 10 μM specific primer mix, 2.0 μL of DNA template mix, 1.0 μL of 20×Eva Green fluorescent dye, and 5 μL of ddH_2_O (Table 1). The reaction conditions are: pre-denaturation at 95°C for 5 min; denaturation at 94°C for 30 s, annealing at 58°C for 30 s, extension at 72°C for 1 min, 35 cycles; final extension at 72°C for 5 min. To determine the optimal reaction system, the aforementioned amplification conditions were applied and used as a basis. By adjusting reaction conditions such as annealing temperature and dNTP concentration, multiplex PCR amplification and HRM analysis were conducted to enhance the experimental efficacy of multiplex PCR-HRM. For the established multiplex PCR-HRM system, those that could produce distinguishable HRM typing curves with stable results were considered valid.

### Quantification and statistical analysis

All quantitative data were analyzed using Microsoft Excel (version 2608). Data are presented as mean ± SD from *n* = 3 independent experiments. The coefficient of variation (CV) was calculated as SD/mean × 100% to assess the reproducibility of measurements and is reported in Table S2.
