# Supplementary material for: An experimental study on HRM combined with multiplex PCR for the identification of single and mixed diatom species
Source: iScience. 2026 Oct 15;29(11):117909. doi: 10.1016/j.isci.2026.117909 (PMC13694805; doi:10.1016/j.isci.2026.117909)
Supplement: Supplementary file 1 [file mmc1.pdf]

iScience, Volume *29*

Supplemental information

An experimental study on HRM combined with multiplex PCR for the identification of single and mixed diatom species

Yunchao Zhou, Yiwei Liu, Bin Cong, Jianhua Chen, and Jianqiang Deng

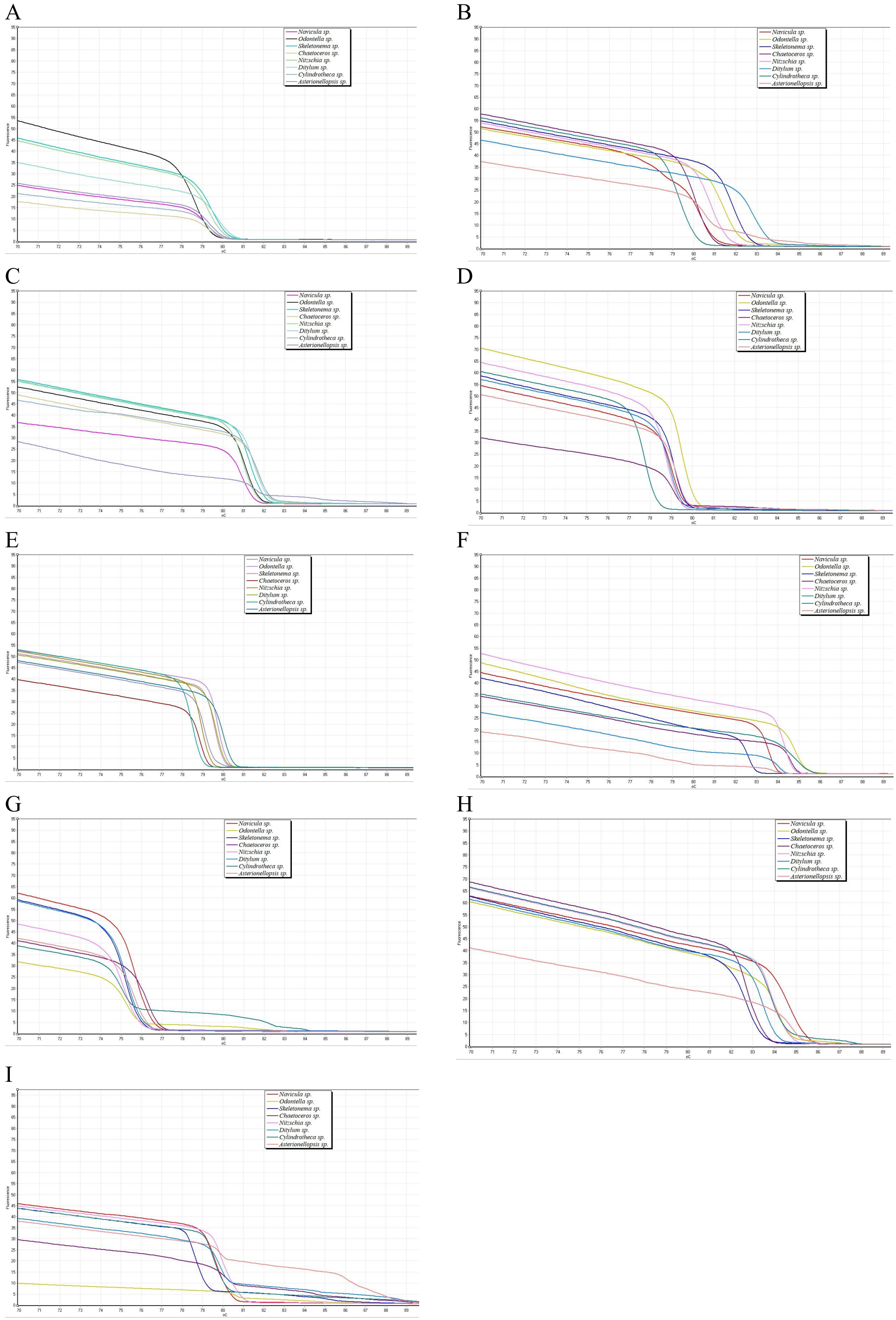

Figure S1. Normalized melting curve graph of HRM results for diatom amplification using different primers, Related to Figure 2 A. Primer *UPA99*, **B.** Primer *18S-V7*, **C.** Primer *PsaA-2*, **D.** Primer *rbcL197*, **E.** Primer *DiatrbcL708*, **F.** Primer *Primer1*, **G.** Primer *ND*, **H.** Primer *28S-191C*, **I.** Primer *18S-333N* could specifically amplify 8 species of marine diatoms. Representative results from 3 independent experiments (n = 3) are shown.

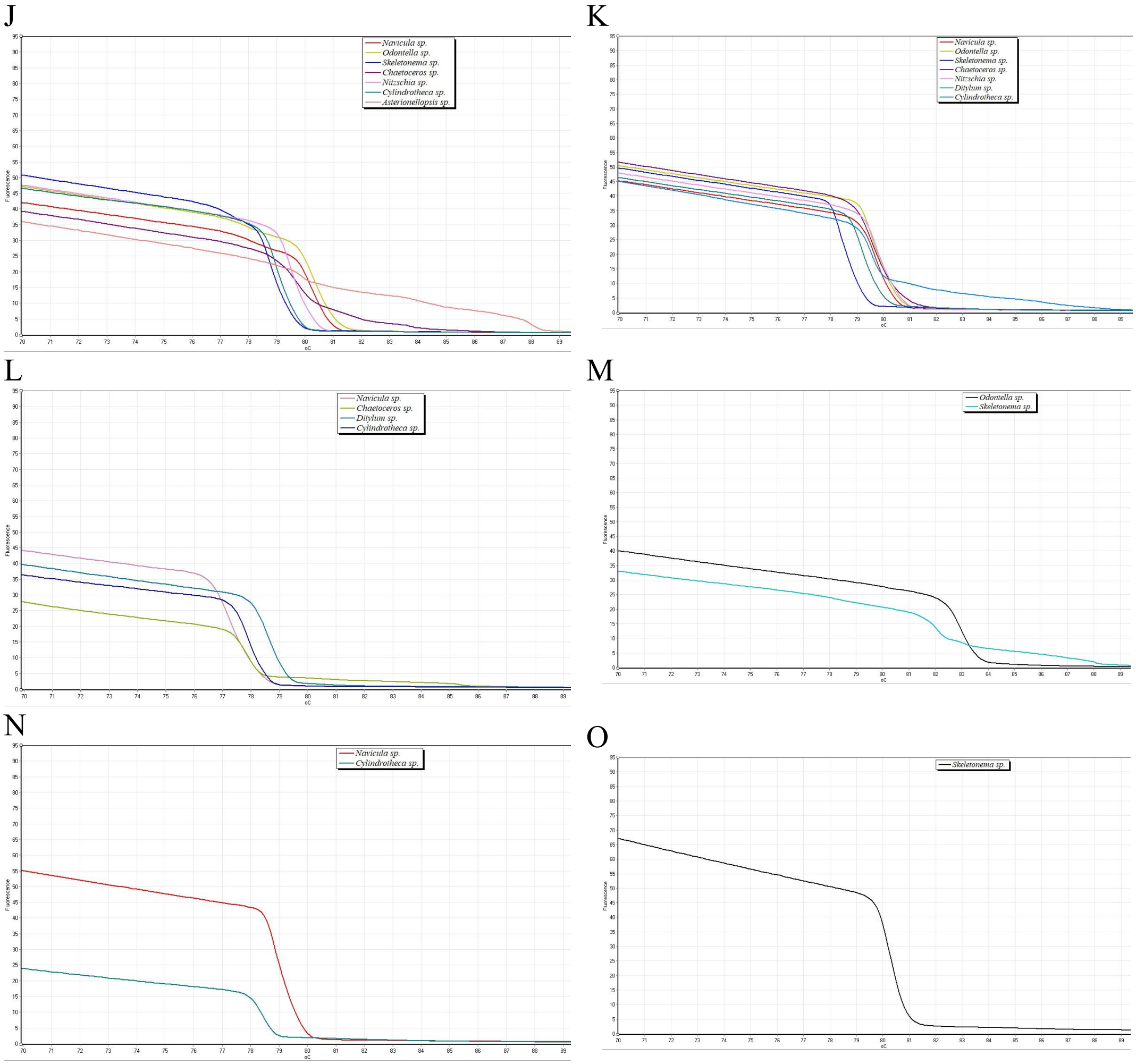

Figure S2. Normalized melting curve graph of HRM results for diatom amplification using different primers, Related to Figure 2 J. Primer *18S-356H*, **K.** Primer *18S-341J* could specifically amplify 7 species of marine diatoms; **L.** Primer *rbcL-256Z* could specifically amplify 4 species of marine diatoms; **M.** Primer *18S-196O*, **N.** Primer *rbcL-N* could only specifically amplify two species of marine diatoms; **O.** Primer *DNGT* could specifically amplify Skeletonema only. Representative results from 3 independent experiments (n = 3) are shown.

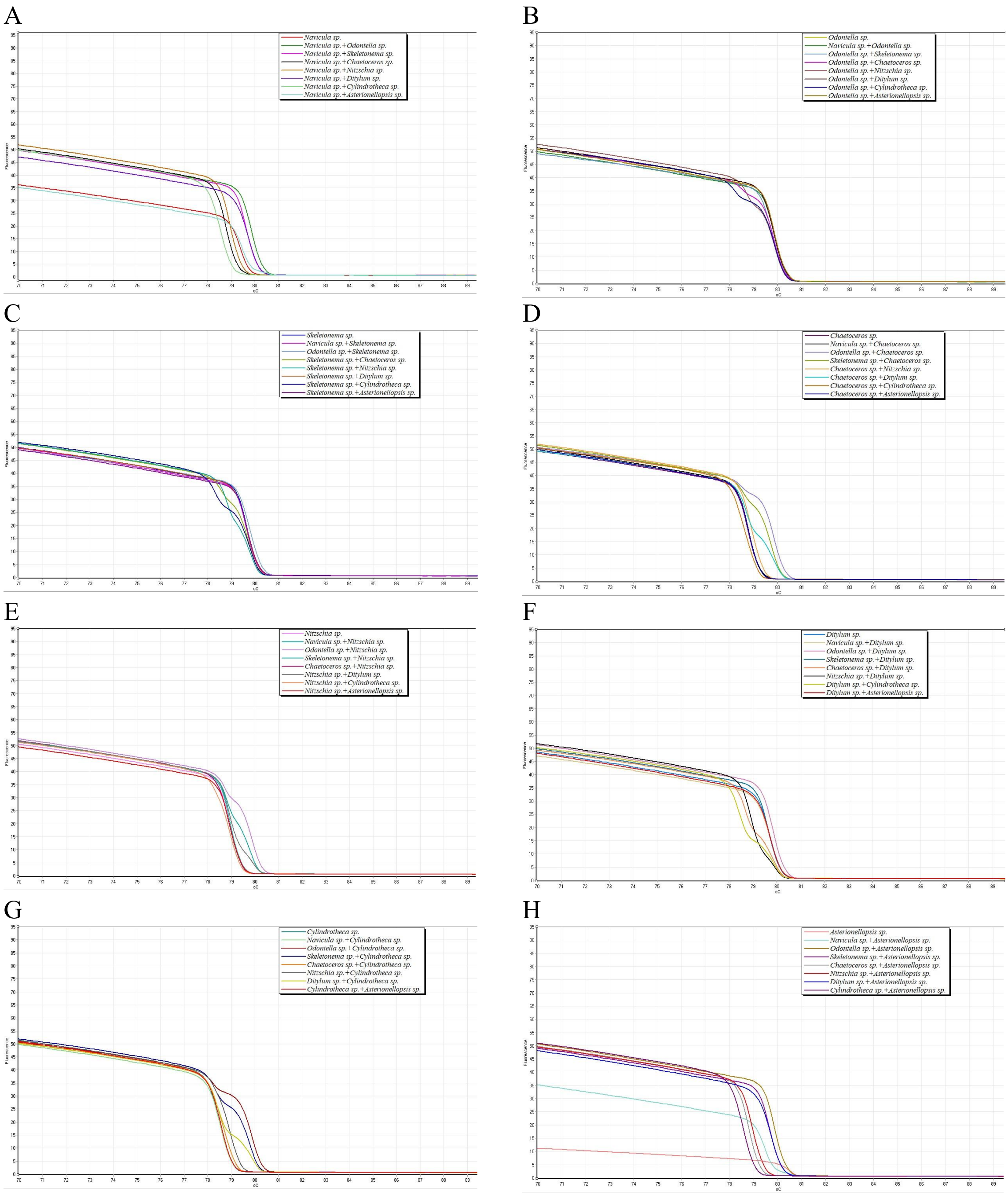

Figure S3 Normalized melting curves of HRM analysis results using primer DiatrbcL708, Related to Figure 3. A~H show the melting curves obtained from pairwise mixtures of *Navicula sp., Odontella sp., Skeletonema sp., Chaetoceros sp., Nitzschia sp., Ditylum sp., Cylindrotheca sp., and Asterionellopsis sp.,* respectively, each mixed with the other seven diatom species (excluding itself). Representative results from 3 independent experiments (n = 3) are shown.

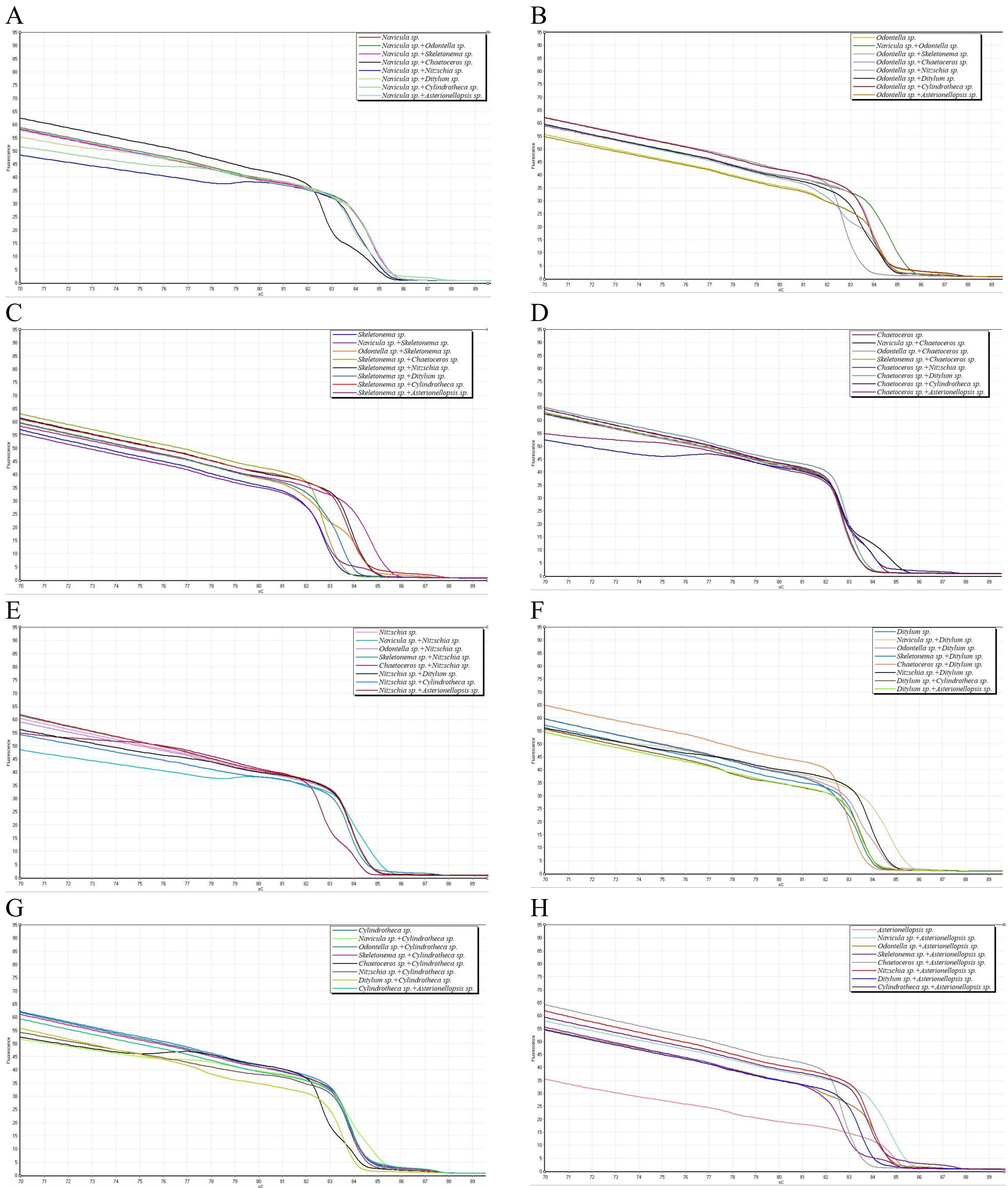

Figure S4 Normalized melting curves of HRM analysis results using primer *28S-191C*, Related to Figure 4. A~H show the melting curves obtained from pairwise mixtures of *Navicula sp.*, *Odontella sp.*, *Skeletonema sp.*, *Chaetoceros sp.*, *Nitzschia sp.*, *Ditylum sp.*, *Cylindrotheca sp.*, and *Asterionellopsis sp.*, respectively, each mixed with the other seven diatom species (excluding itself). Representative results from 3 independent experiments (n = 3) are shown.

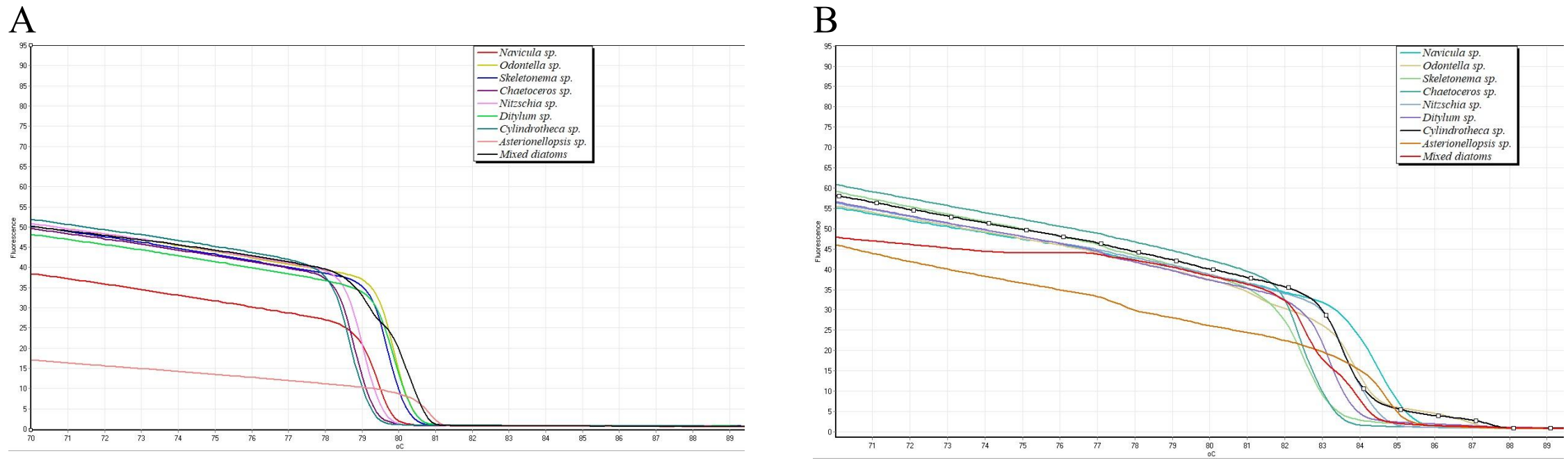

Figure S5 Normalized melting curve plot of the HRM analysis results from eight mixed diatom samples amplified using primers DiatrbcL708 and 28S-191C, respectively, Related to Figure 5. A Melting curve of eight mixed diatom samples amplified with primer *DiatrbcL708*; **B** Melting curves of eight mixed diatom samples amplified with primer *28S-191C*. The curve labeled "Mixed diatoms" represents the mixed sample, while the others are positive controls. Representative results from 3 independent experiments (n = 3) are shown.

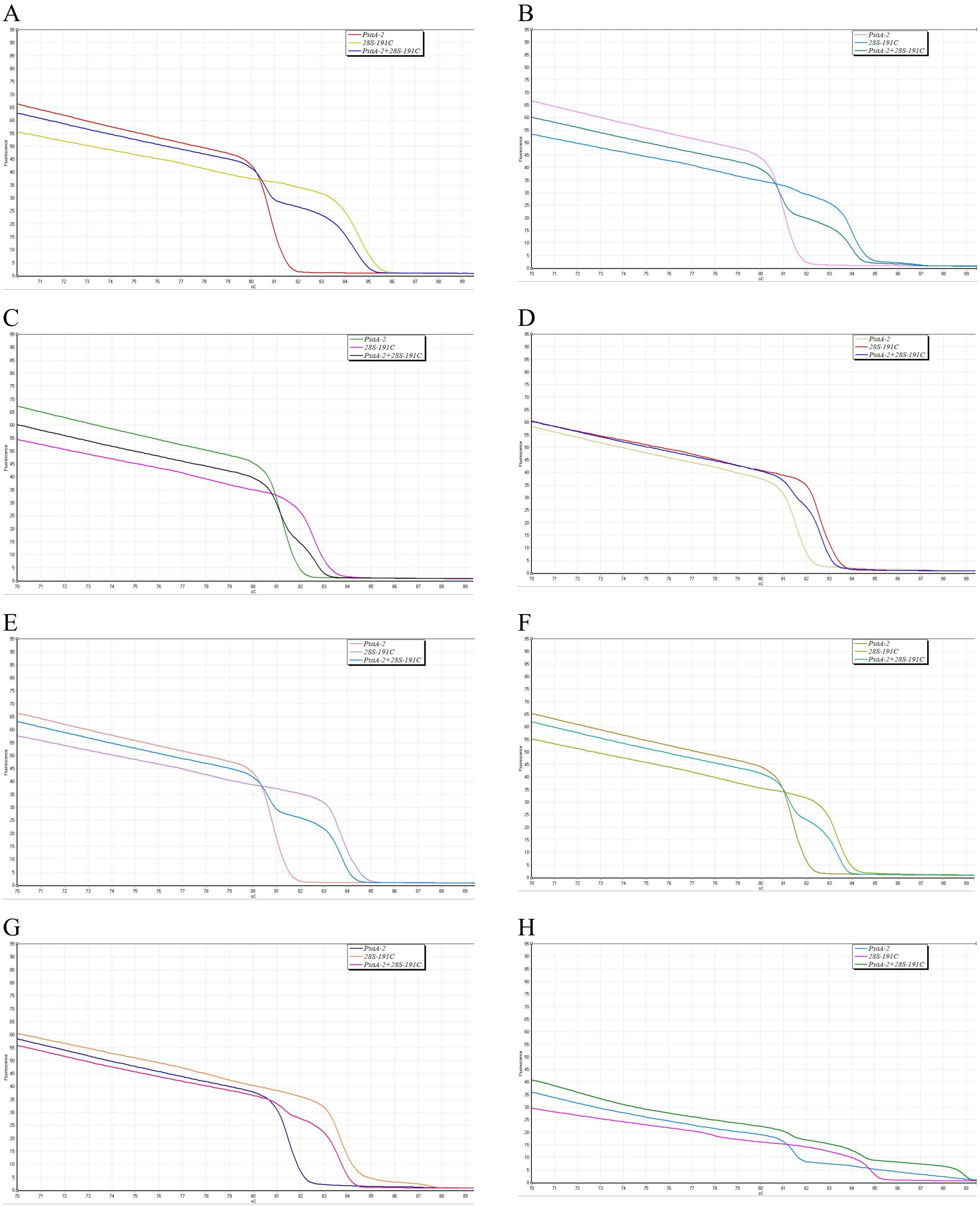

Figure S6. Normalized melting curves of HRM analysis results for eight individual diatom species amplified with mixed primers 28S-191C and PsaA-2, Related to Figure 6. A~H represent *Navicula sp.*, *Odontella sp.*, *Skeletonema sp.*, *Chaetoceros sp.*, *Nitzschia sp.*, *Ditylum sp.*, *Cylindrotheca sp.*, *Asterionellopsis sp.,* respectively. Representative results from 3 independent experiments (n = 3) are shown.

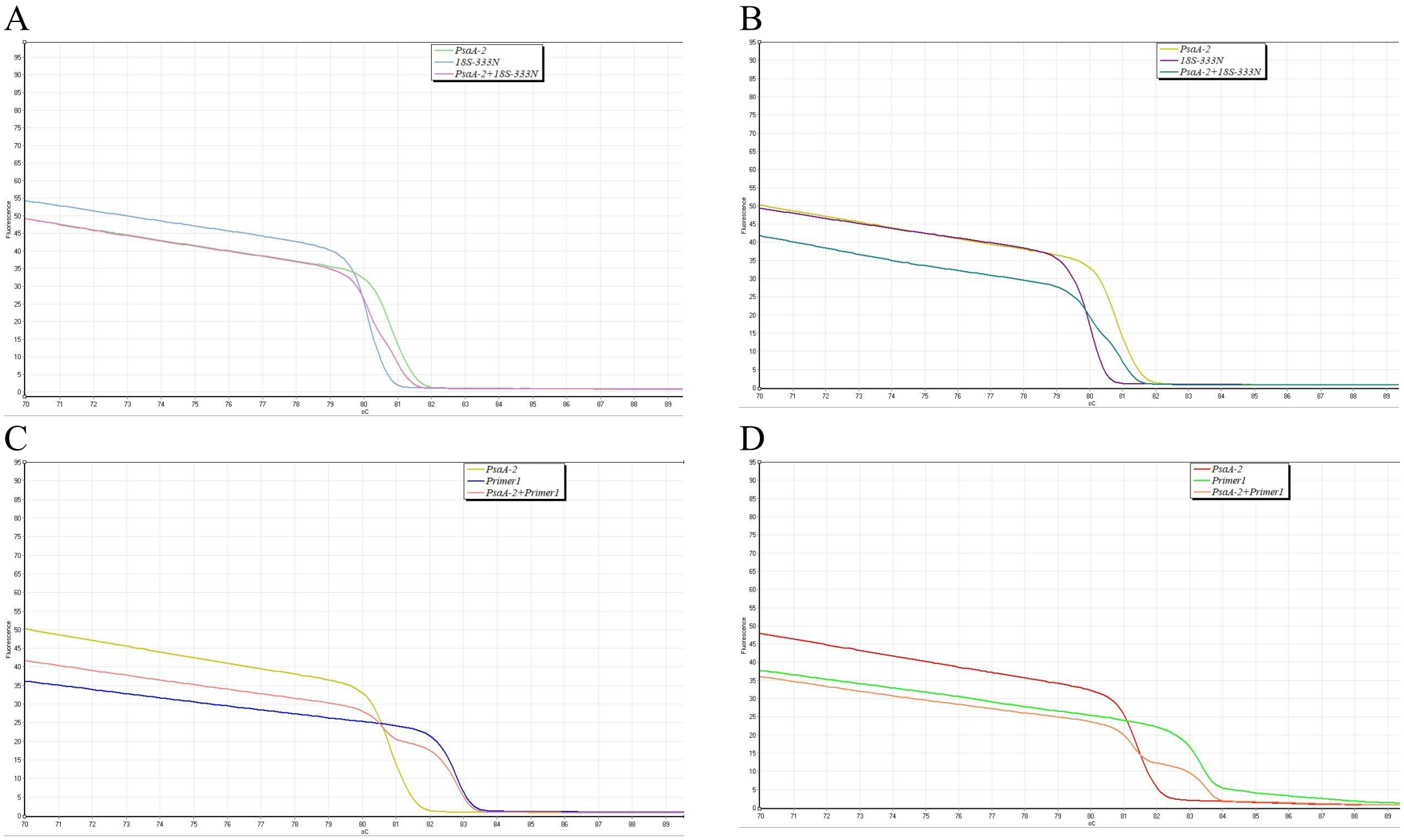

Figure S7. Normalized melting curves of HRM analysis results of individual diatom species amplified with different primer combinations, Related to Figure 7. **A** and **B** show the melting curves obtained by mixed amplification of *Nitzschia sp.* and *Navicula sp.* using primers *PsaA-2* and *18S-333N*, respectively; **C** and **D** show the melting curves obtained by mixed amplification of *Navicula sp.* and *Ditylum sp.* using primers *PsaA-2* and *Primer1*, respectively. Representative results from 3 independent experiments (n = 3) are shown.

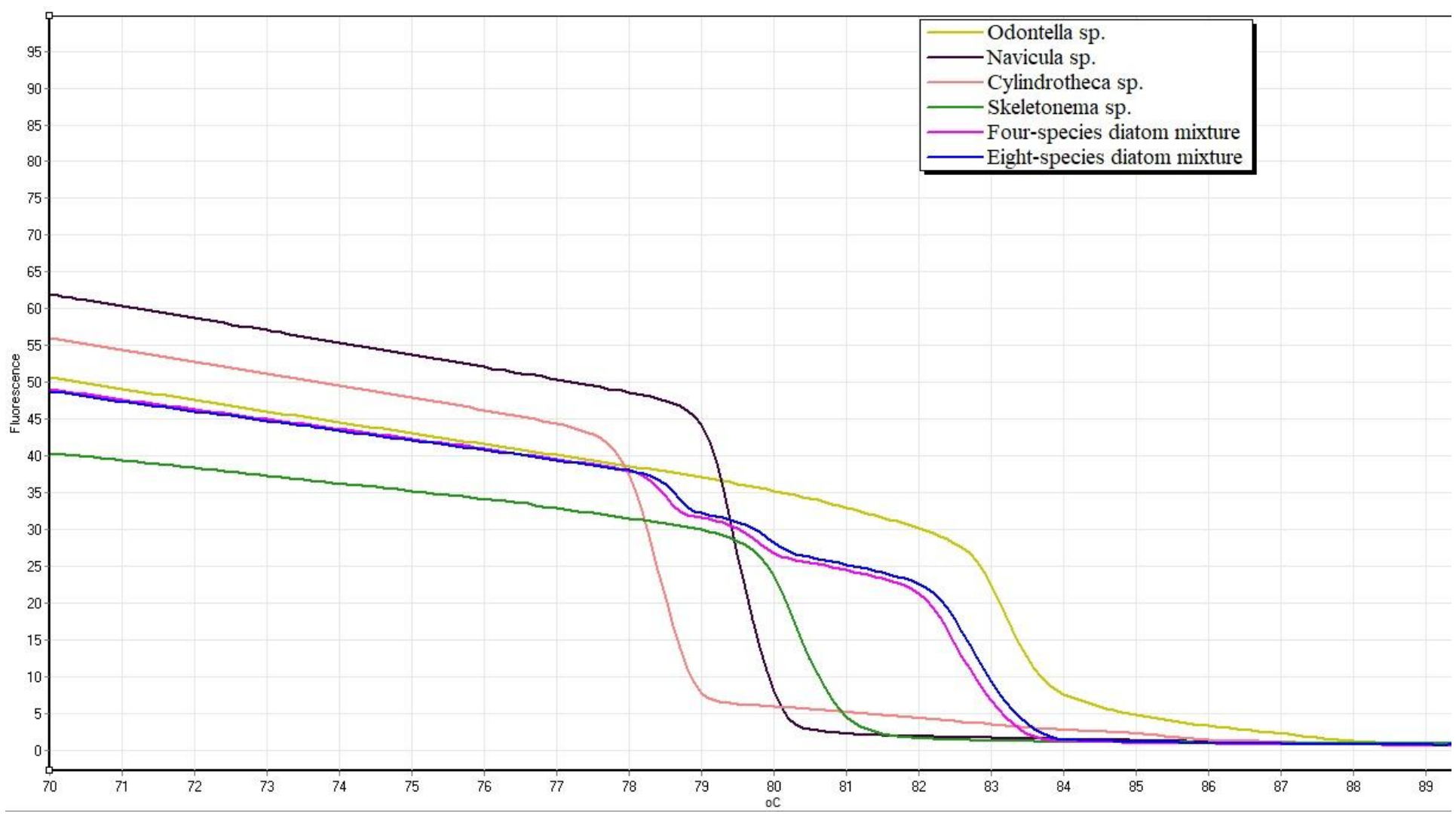

Figure S8. Normalized melting curve analysis of multiplex PCR-HRM amplification products, Related to Figure 8. *Odontella sp., Navicula sp.*, *Skeletonema sp.*, *Cylindrotheca sp.*, a mixture of these four diatom species, and a mixture of eight diatom species were amplified using primers *18S-196O, rbcL-N,* and *DNGT*. Representative results from 3 independent experiments (n = 3) are shown.

Table S1 Accession numbers in GenBank for sequences used for primer design for eight marine diatom species, Related to Table 1

Accession numbers
AY485513.1
HQ396814.1
GQ219684.1
OL780402.1
PQ722531.1
AY485522.1
EU818944.1
KC969979.1
NC_063454.1
NC_067955.1
NC_056371.1
AY485494.1
AY684968.1
MW679509.1
NC_058792.1
AY485453.1
AY485446.1
KC986102.1
FJ432750.1
NC_053621.1
NC_024082.1
LC537470.1
KY863472.1
AJ867277.1
HF679199.1
KX839243.1
NC_061047.1
OR464345.1
AY485526.1
AF374481.2
KC970026.1
EU022312.1
OR666651.1

Table S1 continued

Accession numbers
NC_060383.1
AY485471.1
AY485457.1
KC969907.1
MH716211.1
NC_024082.1
AY485463.1
AY216904.1
KC969865.1
AF417687.1
NC_024080.1

Table S2 Coefficient of variation values of different melting temperatures, Related to Table 2

Primer	*Navicula sp.*	*Odontella sp.*	*Skeletonema sp.*	*Chaetoceros sp.*	*Nitzschia sp.*	*Ditylum sp.*	*Cylindrotheca sp.*	*Asterionellopsis sp.*
UPA99	0.09%	0.18%	0.04%	0.17%	0.20%	0.16%	0.16%	0.23%
18S-V7	0.03%	0.04%	0.02%	0.06%	0.06%	0.06%	0.06%	0.05%
PsaA-2	0.09%	0.09%	0.09%	0.11%	0.10%	0.07%	0.09%	0.15%
rbcL197	0.05%	0.10%	0.11%	0.26%	0.12%	0.06%	0.07%	0.05%
DiatrbcL708	0.18%	0.07%	0.04%	0.07%	0.02%	0.03%	0.04%	0.29%
Primer1	0.45%	0.24%	0.42%	0.39%	0.34%	0.32%	0.37%	0.34%
ND	0.06%	0.14%	0.01%	0.07%	0.07%	0.07%	0.20%	0.37%
DNGT	-	-	0.11%	-	-	-	-	-
18S-333N	0.17%	0.29%	0.17%	0.12%	0.08%	0.15%	0.14%	0.13%
18S-341J	0.07%	0.02%	0.04%	0.20%	0.03%	0.03%	0.24%	-
rbcL-256Z	0.22%	-	-	0.37%	-	0.10%	0.24%	-
rbcL-N	0.04%	-	-	-	-	-	0.34%	-
18S-356H	0.06%	0.11%	0.16%	0.13%	0.22%	-	0.09%	0.24%
28S-191C	0.06%	0.06%	0.07%	0.03%	0.04%	0.03%	0.04%	0.16%
18S-196O	-	0.21%	0.37%	-	-	-	-	-

Note: "-" indicates that the primer does not amplify the corresponding algal species.
